# Unilateral proptosis as the manifestation of euthyroid Graves orbitopathy: A case report

**DOI:** 10.1097/MD.0000000000048416

**Published:** 2026-04-24

**Authors:** Simran Rauniyar, Milan Pokhrel, Sonam Dhenga, Ganesh Chaudhary, Bibek Shrestha

**Affiliations:** a Maharajgunj Medical Campus, Tribhuvan University, Institute of Medicine, Kathmandu, Nepal; b Department of Internal Medicine, Tribhuvan University Teaching Hospital, Kathmandu, Nepal.

**Keywords:** case report, euthyroid, Graves’ orbitopathy, thyroid eye disease, unilateral proptosis

## Abstract

**Rationale:**

To report a case of Euthyroid Graves’ disease with Unilateral eye involvement and its diagnostic and management challenges.

**Patient Concerns:**

A 36-year-old female with left eye proptosis.

**Diagnoses:**

CECT and MRI reported the proptosis of the left eye with thickening of the left extraocular muscles without the involvement of the tendons, without a focal lesion, with mild hyperintensity and mild diffuse enhancement representing unilateral thyroid ophthalmopathy.

**Interventions:**

CECT and MRI for the diagnosis. IV methylprednisolone for the management.

**Outcomes:**

Complete resolution of symptoms by 6 months.

**Learnings:**

1) Although commonly hyperthyroid, patients with Graves’ disease may be euthyroid. 2) Graves’ orbitopathy can affect the unilateral eye. 3) Long-term follow-up and joint endocrine and ophthalmology teamwork required for effective management.

## 1. Introduction

Graves’ disease is an autoimmune condition targeting thyrotropin receptor, resulting in hyperthyroidism, various clinical symptoms, and goiter.^[1]^ Thyroid-associated ophthalmopathy (TAO) or Graves’ orbitopathy (GO) is a common extrathyroidal manifestation of Graves’ disease occurring most commonly in hyperthyroid patients, and to a lesser extent in hypothyroid and euthyroid patients.^[2]^ Existing studies have estimated its prevalence to be 100 to 300/1,00,000 population in Asia,^[3,4]^ while the worldwide prevalence is found to be 7.9% for euthyroidism, 10.36% for hypothyroidism, and 86.2% for hyperthyroidism.^[5]^

Orbitopathy in individuals without thyroid dysfunction or a history of antithyroid treatment is seen in euthyroid Graves’ orbitopathy (EGO) which is a rare condition.^[6]^ EGO has been linked to comparatively lesser degrees of tissue inflammation and muscle involvement than those in hyperthyroidism. Unilateral or asymmetrical presentation is rare.^[7]^ Proliferation of orbital fibroblasts, increased adipogenesis, and expansion of extracellular matrix due to autoimmunity are involved in the pathophysiology, but the exact cause of asymmetry or lateralization is not known.^[8]^

We present a case of a female patient of 36 years of age with left unilateral proptosis with normal thyroid function tests and elevated thyrotropin receptor antibody and MRI findings suggestive of unilateral Graves orbitopathy; highlighting the diagnostic challenge and importance of imaging and multispecialty involvement.

## 2. Case presentation

**Figure 1.**
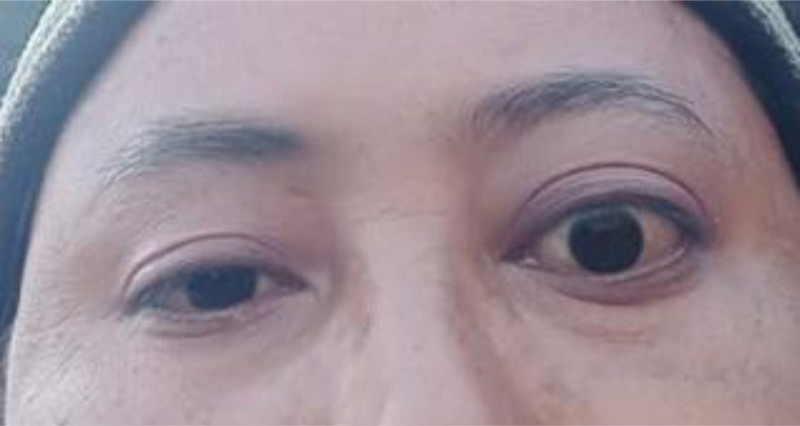
Patient with left proptosis at the time of presentation.

A 36-year-old female presented to the outpatient department of Internal Medicine with the complaints of proptosis of the left eye for 4 months (Fig. 1). It was insidious on onset, progressive, non-pulsatile and not associated with pain or diminution of vision. There was redness and itching but no history of discharge or trauma. She did not complain of any symptoms related to thyroid dysfunction like weight loss, menstrual irregularities, heat/cold intolerance, palpitations, etc. She does not smoke or drink alcohol and is a nonvegetarian. She did not have any remarkable past medical history. There is no family history of thyroid disease.

On examination, she was alert, conscious and cooperative, well oriented to time, place and person. Her vitals were stable. On ocular examination, her extra ocular muscle movements were within range. Visual acquity was 6/6 in bilateral eyes with clinical severity score of 3/7. Hertel’s exophthalmometry of left eye was 23 mm and right was 19 mm. Physical examination including neurological examination was otherwise normal. She did not have any thyroid swelling. Lab investigations for thyroid function are summarized in Table 1.

**Table 1:** Lab investigations.

Parameters	Normal range	At the time of presentation
TRAb	<1.22 IU/L	1.93 IU/L
fT3	2.0–4.4 pg/mL	3.65 pg/mL
fT4	0.93–1.7 ng/dL	1.67 ng/dL
TSH	0.27–4.20 mic.m/L	1.255 mic.m/L

fT3 = free T3, fT4 = free T4, TRAb = thyroid receptor antibody, TSH = thyroid stimulating hormone.

Her hemoglobin, blood sugar, erythrocyte sedimentation rate (ESR), C-reactive protein (CRP), lipid profile, liver function, and renal function were within normal range. Chest x-ray did not show any hilar lymphadenopathy. Sputum acid fast bacilli (AFB) stains and sputum gene Xpert, rheumatoid factor, angiotensin converting enzyme (ACE), and antinuclear antibody (ANA) were negative.

**Figure 2.**
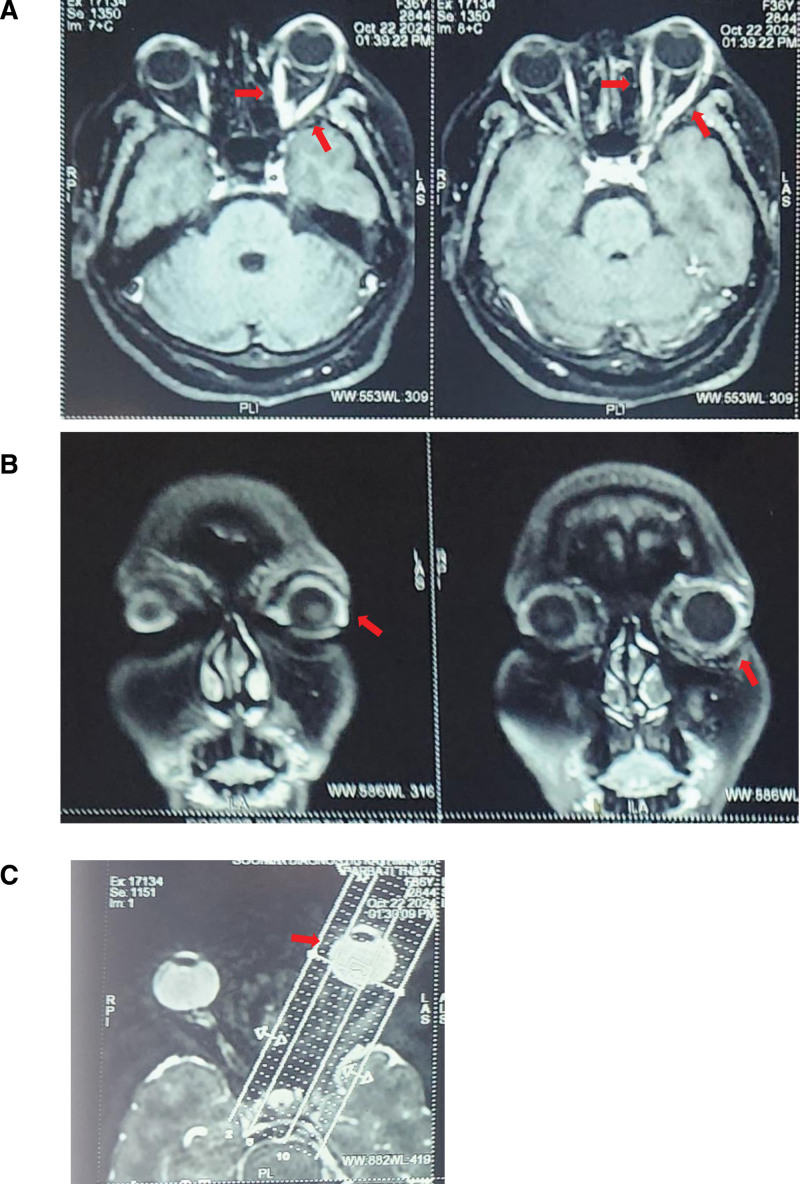
MRI of head and orbit (A and C, axial view; B, coronal view) showing proptosis of the left eye and thickening of the left extraocular muscles without the involvement of the tendons. MRI = magnetic resonance imaging.

USG of neck reported the thyroid gland to be normal in size, echotexture, normal flow and no calcification. Imaging was necessary to exclude other common causes of unilateral proptosis like meningiomas, lymphomas, cavernous carotid fistula, orbital cellulitis, Cushing’s disease, sarcoidosis, pseudotumor cerebri, and primary and metastatic tumors. CECT orbit shows hypertrophied belly of left extraocular muscles with normal tendinous portion and proptosis of left globe; features suggestive of thyroid ophthalmopathy. MRI brain and orbit with contrast (Fig. 2) reported the proptosis of the left eye with thickening of the left extraocular muscles without the involvement of the tendons, without focal lesion, with mild hyperintensity and mild diffuse enhancement representing unilateral thyroid ophthalmopathy. There was no evidence of obvious edema, lesion, or enhancement in the retro-orbital or extra-conal area/fat.

She was diagnosed with euthyroid Graves’ orbitopathy and started on IV methylprednisolone; 500 mg weekly for the first 6 weeks and then 250 mg weekly for the next 6 weeks. As the patient was euthyroid she did not need any antithyroid medications. The patient was followed up till 6 months when her eye symptoms were completely resolved.

## 3. Discussion

GO is a disabling and disfiguring disease of the orbit and unilateral presentation further intensifies it. The prevalence of unilateral disease has been noted to fall between 4.5% and 14%.^[8]^ In our case, the patient presented with unilateral proptosis without any symptoms suggestive of thyroid dysfunction and with an elevated TSH-receptor antibody level of 1.93 IU/L. Age of onset in Graves’ orbitopathy shows bimodal peaks in both sexes, occurring about 5 years earlier in women (40–44 and 60–64 years) than in men (45–49 and 65–69 years).^[7]^ Smoking strongly influences the occurrence and progression of GO while mixed associations between MHC, CTLA-4, PTPN22, interleukins, etc and GO have been identified.^[4]^ Common presentations of GO include lid lag, retraction, proptosis, ophthalmoplegia, conjunctivitis, chemosis, corneal ulceration, and even loss of vision.^[1]^ Symptoms are mostly attributed to orbital fat and extraocular muscle volume within the orbital space.^[1]^ Proptosis and protrusion of the globe results from contained tissues being pushed anteriorly in the incompliant bony orbit.^[1]^ A study dictates the percentages of presenting symptoms in the patients of GO: lid retraction in 57% to 98%, proptosis in 63% to 74%, extraocular muscle involvement in 40% to 60% and optic neuropathy in 5% to 7%.^[3]^ Orbitopathy may be the initial clinical sign of Graves’ disease, and thyroid function tests might not show abnormalities for a prolonged period.^[6]^ Interestingly, a subclinical form of GO can be seen in orbital imaging in over 70% of GO patients highlighting the significance of diagnostic imaging.^[3]^

GO starts with an active phase of inflammation and edema during which anti-inflammatory therapy is effective, followed by a static phase during which fibrosis occurs that limits the tissue recovery.^[3]^ Residual orbitopathy is seen in about 50% of the patients even after 15 years.^[3]^ Despite sharing the thyrotropin receptor (TSH-R) common autoantigen, euthyroid Graves’ disease (EGD) and Graves’ disease are different; the former affects the eye and the orbit while latter affects the thyroid.^[9]^ Pathological changes associated with extraocular muscles and orbital fat are induced by T cell interactions, activation of the PI3K/Akt and cAMP pathways, and insulin-like growth factor.^[9]^ Orbital fibroblasts play a major role in producing glycosaminoglycans and transforming into adipocytes and myofibroblasts.^[9]^ T cell-secreted proinflammatory cytokines contribute to orbital inflammation, tissue remodeling, and muscle enlargement.^[9]^ Laboratory profiles in EGO show an elevated TSH-receptor (TRAb) and thyroid peroxidase antibodies (TPOAb) in most cases.^[10]^ However, 25% of the EGO patients have negative thyroid antibodies. TRAb and TPO might be increased in healthy individuals as well.^[10]^ This adds up to the diagnostic challenge. Common differentials of unilateral eye enlargement are meningiomas, lymphomas, cavernous carotid fistula, orbital cellulitis, Cushing’s disease, sarcoidosis, pseudotumor cerebri, and primary and metastatic tumors; unilateral EGO is rarely thought of.^[7]^ Chronic symptoms, no history of fever, pain or restrictive eye movements rule out orbital cellulitis. The non-pulsatile nature of the eye enlargement rules out carotid-cavernous fistula. Chest x-ray did not show any hilar lymphadenopathy. Sputum AFB stains and sputum gene Xpert, RF, ACE, and ANA were negative. These rule out tuberculosis, sarcoidosis, systemic lupus erythematosus or any rheumatological conditions. Computed tomography or MRI imaging of the orbit is necessary to exclude the differentials mentioned above. Imaging is also needed to diagnose and confirm GO such as extraocular muscle enlargement, adipose tissue expansion, or the sight-threatening complication of dysthyroid optic neuropathy.^[7]^

Both GO and EGO share similar treatment options and EGO patients respond better to treatment due to their milder ophthalmic symptoms and lower clinical activity score.^[1,6]^ European Group on Graves’ Orbitopathy (EUGOGO) guidelines recommend intravenous glucocorticoids as first-line treatment for GO, with 6 weekly infusions of 0.5 g methylprednisolone, followed by six 0.25 g doses.^[1]^ Combining systemic steroids with orbital radiotherapy has greater and faster improvement.^[7,11]^ A combination of glucocorticoids and mycophenolate mofetil has shown improved long-term outcomes in real-world patients. In glucocorticoid-resistant cases, other immunomodulatory treatments include rituximab, tocilizumab, and the FDA-approved (2020) teprotumumab.^[1]^ New research has identified potential therapies, including co-stimulation inhibitors like abatacept and alefacept, which may block T and B cell activation. Rituximab, targeting B cell maturation, could also be helpful due to the role of TRAbs and IGF-1r autoantibodies in GO development.^[9]^

Table 2 enlists the similarities, key differences and unique features of our case as compared to existing literature. While bilateral cases are common, unilateral presentation in Grave’s disease is rare, even in hyperthyroid conditions. Bhattarai et al reports a case of hyperthyroidism presenting with unilateral proptosis, without any visual disturbances, and with other symptoms of hyperthyroidism like weight loss, menstrual irregularities, and palpitations.^[14]^ Likewise, cases of EGO have been reported in literature where the patients had unilateral proptosis and responded well to the steroid therapy.^[12,13]^ In contrast, Tabasum et al reports a case of EGO with negative thyroid antibodies which ultimately developed thyroid dysfunction 24 months after the initial presentation with eye disease, which is an established sequelae in some patients.^[7]^ This points out the potential natural evolution of Graves’ disease and demands long-term follow-up of EGO patients with joint ophthalmic and endocrine evaluation.

**Table 2:** Comparison of unilateral Graves’ orbitopathy cases with our patient, highlighting similarities, differences, and unique features.

Reference/year	Case summary	Similarities with our case	Key differences	Unique features of our case and clinical reflections
Arslan et al 2009^[12]^	A 43-yr-male, smoker patient presented with decreased vision in right eye, a normal TFT, an elevated TRAb and an MRI finding in right eye consistent with Graves’ orbitopathy; diagnosed of EGO with optic neuropathy. Vision was restored upon systemic methylprednisolone administration	In both the cases, the patients had unilateral eye involvement, a normal TFT and an elevated TRAb. Both the cases showed good response to systemic corticosteroids	While this case highlights the severe disease-optic neuropathy as initial presentation in EGO, milder presentation like that of our patient should not be undermined and early imaging should be done to recognize the disease in time. Further, our patient was a nonsmoker, that might address the milder presentation	Painless, unilateral proptosis without vision loss/decrement can still be an early manifestation of Graves disease in euthyroid patients and needs early imaging for timely identification and treatment
Michigishi et al, 2024^[13]^	A 39-yr-old male presented with gradual, painless, left eye proptosis, normal TFT and an elevated TRAb and Tg. He also had an autonomously functioning thyroid papillary carcinoma. His MRI findings of the orbit led to diagnosis of Graves orbitopathy. Subtotal thyroidectomy was done for the nodule and the orbitopathy responded well to IV methylprednisolone	Both the cases had unilateral presentation and were euthyroid. Imaging confirmed the diagnosis and response to systemic corticosteroid was good	Our patient had isolated autoimmune EGO with structurally normal thyroid gland, highlighting a distinct autoimmune pathogenic mechanism compared to Michigishi et al, where an association of autonomously functioning thyroid carcinoma complicates the diagnosis, requiring T3 suppression tests, scintigraphy, autoradiography, etc	Isolated, mild cases of unilateral EGO in euthyroid patient have a straightforward diagnosis based on positive TRAb and MRI. Concurrent associations like thyroid carcinoma may overshadow the orbital presentation of EGO and add to the diagnostic complexity
Bhattarai et al 2023^[14]^	A 25-yr-old female presented with progressive right eye bulging and features of hyperthyroidism (weight loss, menstrual irregularities, palpitations). Her TFT showed hyperthyroidism and her TRAb was also elevated. MRI confirmed the diagnosis of hyperthyroid Graves’s disease with Graves’s orbitopathy. She responded well to IV methylprednisolone and antithyroid medication-methimazole	Both the cases had unilateral presentation and an elevated TRAb, indicating autoimmune pathology	Our patient was euthyroid and steroid was adequate for symptom resolution; while Bhattarai et al reported unilateral proptosis in hyperthyroid setting. Treatment required combination of systemic steroids and antithyroid medication due to hyperthyroid status	Imaging is more crucial in cases of unilateral proptosis with euthyroid profile as the diagnosis depends entirely on the MRI findings
Tabasum et al 2016^[7]^	A 66-yr-old female presented with right eye pain and diplopia, normal TFT and negative TRAb and TPOAb. CT and MRI confirmed the diagnosis of GO with asymmetrical involvement (right eye more than the left). She underwent surgical recession of inferior rectus with complete resolution of her symptoms. After 24 mo of initial presentation, she became hyperthyroid and developed positive TRAb and TPOAb. She responded to treatment with thionamides and remained euthyroid	Unilateral/asymmetrical proptosis and imaging findings leading to diagnosis of Graves’ orbitopathy is common in both the cases	Our case had a positive TRAb right from the time of presentation unlike in Tabassum et al where TRAb was initially negative leading to diagnostic uncertainty. Additionally, the modality of treatment was different due to severe symptoms like diplopia and orbital pain. The patient eventually developed hyperthyroidism	Despite many differential diagnoses of unilateral proptosis, a positive TRAb directs the case towards GO. Cases with Milder symptoms and lower clinical severity score respond well to medical therapy with systemic steroids while those with severe symptoms like diplopia require surgery. Long-term follow-up is crucial to identify changes in the thyroid profile

CT = computed tomography, EGO = euthyroid Graves’ orbitopathy, GO = Graves’ orbitopathy, MRI = magnetic resonance imaging, TFT = thyroid function test, Tg = thyroglobulin, TPOAb = thyroid peroxidase antibody, TRAb = thyroid receptor antibody.

## 4. Conclusion

The unilateral presentation and euthyroid state of Graves’ orbitopathy present a diagnostic challenge, emphasizing the need for imaging for timely identification to prevent complications and progression to more severe stages. Long-term follow-up is crucial for patients with EGO, involving joint ophthalmic and endocrine evaluations, as a euthyroid state may evolve into hyperthyroidism, and unilateral cases can progress to bilateral involvement. Early management can significantly improve outcomes.

## Author contributions

**Conceptualization:** Simran Rauniyar, Milan Pokhrel.

**Data curation:** Simran Rauniyar, Milan Pokhrel.

**Formal analysis:** Simran Rauniyar, Milan Pokhrel.

**Investigation:** Sonam Dhenga, Ganesh Chaudhary, Bibek Shrestha.

**Methodology:** Simran Rauniyar, Milan Pokhrel.

**Project administration:** Simran Rauniyar, Milan Pokhrel.

**Supervision:** Sonam Dhenga, Ganesh Chaudhary, Bibek Shrestha.

**Validation:** Sonam Dhenga, Ganesh Chaudhary, Bibek Shrestha.

**Visualization:** Simran Rauniyar, Milan Pokhrel.

**Writing – original draft:** Simran Rauniyar, Milan Pokhrel.

**Writing – review & editing:** Simran Rauniyar, Milan Pokhrel.
